# Inequality in hospitalization due to non-communicable diseases in Sweden: Age-cohort analysis of the Uppsala Birth Cohort Multigenerational Study

**DOI:** 10.1016/j.ssmph.2021.100741

**Published:** 2021-01-21

**Authors:** Dawid Gondek, George B. Ploubidis, Muhammad Zakir Hossin, Menghan Gao, David Bann, Ilona Koupil

**Affiliations:** a Centre for Longitudinal Studies, UCL Institute of Education, University College London, United Kingdom; b Department of Public Health Sciences, Karolinska Institutet, Stockholm, Sweden; c Department of Public Health Sciences, Stockholm University, Stockholm, Sweden

**Keywords:** Trends in hospitalization, Expansion of morbidity, Sweden, Socioeconomic inequality, Chronic health

## Abstract

We aimed to investigate cohort differences in age trajectories of hospitalization due to non-communicable conditions, and if these varied by paternal socioeconomic position. We used the Uppsala Birth Cohort Multigenerational Study—including virtually complete information on medical diagnoses.

Our sample constituted 28,448 individuals (103,262 observations). The outcome was five-year prevalence of hospitalization due to major non-communicable conditions in 1989–2008. The exposures were age (19–91), year-of-birth (1915–1929; 1938–1972), gender (man vs woman), and parental socioeconomic position (low, medium, and high). We used multilevel logit models to examine associations between exposures and the hospitalization outcome.

Younger cohorts had a higher prevalence of hospitalization at overlapping ages than those born earlier, with inter-cohort differences emerging from early-adulthood and increasing with age. For instance, at age 40 predicted probability of hospitalization increased across birth-cohorts—from 1.2% (born in 1948-52) to 2.0% (born in 1963-67)—whereas at age 50 it was 2.9% for those born in 1938-42 compared with 4.6% among participants born in 1953-57. Those with medium and low socioeconomic position had 13.0% and 20.0% higher odds of experiencing hospitalization during the observation period, respectively—when age, year-of-birth and gender were accounted for.

We found that no progress was made in reducing the socioeconomic inequalities in hospitalization across cohorts born between 1915 and 1972. Hence, more effective policies and interventions are needed to reduce the overall burden of morbidity—particularly among the most vulnerable.

## Highlights

• What is already known on this subject?
• The evidence on trends in morbidity in Sweden is mainly cross-sectional and focused on individual conditions.
• Rates of various indicators of morbidity (e.g. poor mobility, psychological distress, disability) have increased over time.
• What this study adds.
• Successively younger birth cohorts had a higher prevalence of hospitalization, with differences emerging in early-adulthood.
• Those in medium and low parental socioeconomic position (vs high) had 13% and 20% higher odds of hospitalization.
• No progress was made in reducing the socioeconomic inequalities across cohorts born between 1915 and 1972.

## Introduction

Life expectancy at birth has increased by six years in Sweden and eleven years across the members of the Organization for Economic Co-operation and Development (OECD) in the last five decades (Health at a Glance, 2017). Nonetheless, growing evidence suggests that this increase in the lifespan has not translated into longer periods of life free of morbidity (Gondek et al., 2019; Salomon et al., 2012). Due to population ageing, non-communicable morbidity is projected to rise further—presenting policymakers with a challenge related to future healthcare policy, allocation of resources (Erler et al., 2011) and predicting trends in the workforce (Crimmins et al., 1999). Those with lower socioeconomic status or living in poorer areas appear to suffer from higher rates of chronic conditions compared with their better-off counterparts (Fors et al., 2008; Fritzell et al., 2007; Kunst et al., 2005; Lahelma et al., 2002; Meinow et al., 2015). This is despite Sweden having been particularly determined to reduce the health gap between rich and poor, making it a central objective of public health and social policy agendas since 1980s (Lundberg, 2018). An important step in the development of health policies and interventions is to produce high-quality evidence on how changing socioeconomic and policy contexts have affected health over time, and if these effects varied across socioeconomic groups.

Currently, the evidence on secular trends and the socioeconomic gap in non-communicable morbidity in Sweden is mainly cross-sectional, limited to comparing age-standardized rates of individual conditions over time across all ages combined (Andersson et al., 2015; Paren et al., 2014) or in older age exclusively (Fors et al., 2008; Meinow et al., 2015; Parker & Thorslund, 2007). These studies have shown worsening in various self-reported health domains in the last three decades—when ageing was accounted for—including mobility, psychological distress, disability, chronic conditions as well as objectively-measured physical capacity, lung function, and vision (Fors et al., 2008; Meinow et al., 2015; Parker & Thorslund, 2007). As in other high-income countries, the speculative reason for greater rates of morbidity is an improved survival among the older population with non-communicable health problems (Meinow et al., 2015; Rosen & Haglund, 2005). However, contrary evidence also exists, suggesting an increase in healthy life expectancy (Salomon et al., 2012) and years free from disability and mobility problems from early 1990s until early 2010s in both elderly men and women (Sundberg et al., 2016).

Evidence consistently points towards a health gradient according to social class, education or income (Fors et al., 2008; Fritzell et al., 2007; Kunst et al., 2005; Lahelma et al., 2002; Meinow et al., 2015)—with the differences remaining stable over time in the last few decades (Fritzell et al., 2007; Hu et al., 2016; Kunst et al., 2005; Lahelma et al., 2002). However, the evidence on cohort differences in age trajectories of morbidity—to our best knowledge—is virtually absent in Sweden. There are a few studies, mainly conducted in Great Britain, which explored cohort trends in age trajectories of body mass index, blood pressure and frailty (Bann et al., 2017; Marshall et al., 2015; Wills et al., 2011). Such studies are important as they allow for identifying life stages when the differences across cohorts start to emerge. In addition, life course studies conducted across different birth cohorts allow for disentangling age and cohort effects, which is not possible in cross-sectional studies due to exact collinearity of age and year-of-birth.

None of the life course studies, however, examined trends in non-communicable diseases and hospitalization. Our research contributes to the literature by focusing on inpatient hospital admissions, which indicate direct demands on health services and are also closely associated with other health measures, such as self-reported health, all-cause mortality (Lofqvist et al., 2014) and quality of life (Farkas et al., 2010; Fleury et al., 2014). Inpatient care constitutes one-fourth of total health expenditure in Sweden, mainly driven by cancer and heart diseases (OECD/EU, 2016). A large proportion of inpatient admissions, for such conditions as asthma or diabetes, could be managed in primary care or community settings, which would reduce costs and improve the effectiveness of health care (OECD/EU, 2016; Pezzin et al., 2018). Hence, hospital admissions are a particularly useful outcome to monitor over time. Studying cohort effects in admissions helps to project future demands. Considering age at which cohort differences emerge is also important as health-care spending increases after age 50 and escalates after age 70 (Kelly et al., 2016).

We also examined whether cohort differences in age trajectory of hospitalization varied by parental socioeconomic position (SEP). Higher rates of hospital admission among disadvantaged socioeconomic groups have been found in Sweden (avoidable hospitalization) (Lofqvist et al., 2014), New Zealand (for general and psychiatric admissions) (Barnett & Lauer, 2003), Amsterdam (for psychiatric admissions), Norway (Carlsen et al., 2007), and Australia (Brameld & Holman, 2005), with trends being less clear in Canada (Asada & Kephart, 2007), Italy (Antonelli-Incalzi et al., 2007), and the USA (Taylor et al., 2006). None of the above studies used childhood socioeconomic indicators. Childhood socioeconomic circumstances are important health determinants as they have been found to be associated with adult non-communicable health, independently of adult SEP (Birnie et al., 2011), and their negative health effects are likely to accumulate over the life course (Ben-Shlomo & Kuh, 2002). Using parental SEP also has a methodological advantage, as it is less likely than adult measures of SEP to suffer from reverse causality (Warren, 2009). Currently, the evidence in Sweden is limited to adult socioeconomic indicators (Fors et al., 2008; Fritzell et al., 2007; Hu et al., 2016; Kunst et al., 2005; Lahelma et al., 2002; Meinow et al., 2015). Finally, we explored gender effects in cohort and socioeconomic differences in hospitalization.

We used the Uppsala Birth Cohort Multigenerational Study (UBCoS Multigen), which is linked to the general population and health registers in Sweden, providing virtually complete information on medical diagnoses and family links. This allowed us to study secular trends in age trajectories of hospitalization across two generations (over the period of 1989–2008)—among those born in 1915–1929 and their children born in 1938–1972, ranging in age from 19 to 91. Due to recent evidence on the increase of burden due to non-communicable morbidity in Sweden and other high-income countries (Andersson et al., 2015; Fors et al., 2008; Gondek et al., 1946; Meinow et al., 2015; Paren et al., 2014; Parker & Thorslund, 2007; van Oostrom et al., 2016), we hypothesized rising rates of hospitalization across cohorts. Those in lower socioeconomic position were hypothesized to have greater rates of hospitalization, with the socioeconomic gradient remaining stable over time (Fritzell et al., 2007; Hu et al., 2016; Kunst et al., 2005; Lahelma et al., 2002).

## Methods

### Study population

**Fig. 1:**
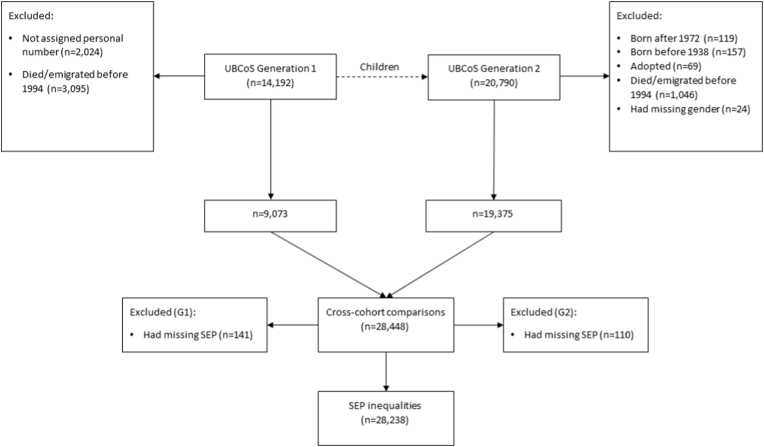
Flow chart of sample selection.

We used data from the UBCoS Multigen (Koupil, 2007; Koupil & Goodman, 2011) – the cohort of 14,192 men and women, born in the Uppsala University Hospital (Uppsala, Sweden) between 1915 and 1929 (G1), and their children (G2) identified through the Multi-Generational Register. Among the members of G1, 12,168 were living in Sweden in the late 1940s, hence they received unique personal identification numbers (Ludvigsson et al., 2009), which remains unchanged and allows for the linkage across national registers. The study was approved by the Regional Ethics Board in Stockholm. G1 is nationally representative of Sweden in terms of infant mortality and fertility (Goodman & Koupil, 2009), with a higher proportion of births to single mothers (Rajaleid et al., 2008) and infants from urban areas (Goodman et al., 2012). Fig. 1 depicts the selection process of the study population. Our sample was limited to G1's biological children born between 1938 and 1972—as there were too few individuals beyond these birth cohorts. We only included participants who contributed to at least one 5-year-interval observation period. Hence, those who died or emigrated from Sweden before 1994, which was the end of the first 5-year-interval of the observation period, were excluded (n = 4141) (see Supplementary Table 1 for characteristics of excluded individuals). Those with missing gender information (n = 24) were also excluded. The final sample in our study constituted 28,448 individuals (28,238 in models with SEP variable) with 103,262 observations (102,571 in models with SEP variable). Being a man and having medium or low SEP were predictive of being excluded or not contributing observations to the entire observation period.

### Variables

Our outcome of interest was any hospitalization due to a major non-communicable condition (see Table 1 for details) within four 5-year intervals (1989–1993, 1994–1998, 1999–2003, 2004–2008). These conditions were selected as they constitute the key contributors to the overall burden of morbidity (Salomon et al., 2012), and they are preventable and socially structured (Cockerham et al., 2017). The outcome was ascertained from the Swedish National Patient Register, which has complete coverage of all public and private inpatient care since 1987 (Ludvigsson et al., 2011). Cases were identified as individuals with a recorded primary or secondary diagnosis with the corresponding International Classification of Disease Ninth and Tenth Revisions (ICD-9/10) codes in the register (Tonelli et al., 2015) (see Supplementary Table 2 for details). The exposures of interest were age (19–91), year-of-birth (1915–1972), gender (man vs woman), and parental socioeconomic position (SEP). Parental SEP was categorized as “high”, “medium”, and “low”. It was derived, using the Swedish socioeconomic classification (Statistics Sweden, 1984), from the fathers’ or mothers’ occupations recorded in archived obstetric records, school records or Census 1930 for G1 and the Population and Housing Census 1960 for G2.

**Table 1:** Description of variables used in the analysis.

Variable	Description
**Outcome**	
Hospitalization due to non-communicable morbidity	A binary indicator (0 = Not hospitalized, 1 = Hospitalized) of any public and private inpatient hospitalization, within four 5-year intervals (1989–1993, 1994–1998, 1999–2003, 2004–2008), due to: depression, asthma, cerebrovascular disease, chronic kidney diseases, cirrhosis, chronic obstructive pulmonary disease, dementia, diabetes, heart failure, cancer, hypertension, ischemic heart disease, migraine, Parkinson's disease, rheumatoid arthritis. Information on hospitalization was ascertained from the Swedish National Patient Register, which includes complete information on the public and private inpatient care utilization since 1987, using ICD-10 and equivalent ICD-9 codes (see Supplementary Table 2 for details).
**Exposures**	
Age	A continuous variables ranging from 19 to 91. Age was derived by subtracting mid-year within each 5-year interval from year-of-birth (e.g. those born in 1962 would have age of 29 in the observation period of 1989–1993, 34 in 1994–1998, 39 in 1999–2003 and 44 in 2004–2008) (see Supplementary Figure 1 for graphical representation of the data).
Year-of-birth	A continuous variable ranging from 1915 to 1929 (G1) and 1938–1972 (G2). Year-of-birth was centered on its grand mean.
Gender	A binary indicator (0 = Man, 1 = Woman). Sample included 14,234 (50.04%) men and 14,214 (49.96%) women.
Parental socioeconomic position	A categorical variable (0 = High, 1 = Medium, 2 = Low), was derived based on the approach used by Sidorchuk, Goodman, and Koupil (2018)(48):
	• Parental SEP for G1 was based on father's occupation, if available; otherwise on mother's occupation and categorized into (Health at a Glance, 2017) high: higher/intermediate non-manuals, academic professionals; (Salomon et al., 2012) medium: the self-employed, farmers and lower non-manuals; and (Gondek et al., 2019) low: skilled/unskilled manuals. Data was retrieved from archived obstetric and school records and Census 1930.
	• Parental SEP for G2 was based separately on father's and mother's occupation and categorized as (Health at a Glance, 2017) high: higher/intermediate non-manuals, self-employed in academic professions; (Salomon et al., 2012) medium: the self-employed in industry, trading, transport or service with employees, farmers with employees, lower non-manuals and persons at the military service; and (Gondek et al., 2019) low: skilled/unskilled manuals, self-employed in industry, trading, transport or service without employees, farmers without employees, students, persons with unidentified occupation and others. The highest parental social class was defined by comparing maternal and paternal occupations. Data was obtained from the Population and Housing Census 1960.
	Sample included 9616 (34.05%) people in high SEP, 6453 (22.85%) in medium SEP and 12,169 (43.09%) in low SEP.
**Random (structural) variables**	
Observation	103,262 observations (102,571 in models with SEP variable).
Individual	28,448 individuals (28,238 in models with SEP variable).
Family	16,203 families (16,027 in models with SEP variable); the variable was created by linking siblings within generation 2 with their parents (G1) and siblings across G1.

### Analysis

Data were set up as an accelerated longitudinal study, with 1915–1929 (G1) and 1938–1972 (G2) birth cohorts observed at 5-year-interval periods (1989–1993, 1994–1998, 1999–2003, 2004–2008). In such a set-up of registry-based data, we can observe cohort-specific age trajectories of hospitalization over the overlapping 20-year study period (Supplementary Figure 1). This allows for comparing the level and rate of change in hospitalization at equivalent ages, but across individuals born at different points in time. We used a multilevel growth curve framework in our analyses—with a logit link function due to a binary outcome.

This framework allows for modelling data that are unbalanced in time—where some individuals do not contribute data during the entire observation period, in this case, due to death or emigration (Raudenbush & Bryk, 2002). It also accounts for hierarchical dependency of observations (level 1) within individuals (level 2)—with age becoming an observation-level variable (Suzuki, 2012). We extended this model by including family identifier as a third hierarchical level, in order to account for dependency of individuals within the same families across two generations. Hence, our models had three hierarchical levels: observations (n = 102571-103,262; level 1) nested within individuals (n = 28,238-28,448; level 2) nested within families (n = 16,027-16,203; level 3)—included as random intercepts (see Supplementary Figure 2).

All exposure variables and their interactions were specified in fixed part of the model. We did not explicitly modelled period effects, as the focus of our analyses was on differences due to year-of-birth in age trajectories. If periodical changes were at play, they would result in cohort differences in the age trajectory of hospitalization as birth cohorts vary by age in any historical moment. Hence, inter-cohort variations due to period effects were captured by controlling for year-of-birth and age*year-of-birth interaction (Yang, 2007). All models were estimated using Markov chain Monte Carlo (MCMC) in MLwiN v3.03 (Charlton et al., 2019) with the runmlwin command in Stata 15 (Leckie & Charlton, 2013).

### Cohort differences in hospitalization

We tested for inter-cohort differences in age trajectories of hospitalization, by fitting a model including age and year-of-birth polynomials as far as significant up to a cubic term (p < 0.05), alongside an age*year-of-birth interaction. Year-of-birth and age were centered on its grand mean to alleviate the interference of the nonessential multicollinearity (Iacobucci et al., 2016), allowing for estimating non-linear age and year-of-birth polynomials. Subsequently, we examined if men or women experienced any inter-cohort differences in hospitalization by adding to the model gender variable and gender*year-of-birth interaction.

### Socioeconomic inequalities in the rate of hospitalization

Subsequently, we examined time and gender effects in socioeconomic inequalities in hospitalization. We fitted a model including age and year-of-birth polynomials as far as significant up to a cubic term (p < 0.05), alongside an age*year-of-birth interaction, gender and SEP variables as well as SEP*year-of-birth, and SEP*age. SEP*year-of-birth, SEP*age interactions were tested one-by-one at the significance level of p < 0.05. The analysis was also conducted using the highest recorded education at age 19 or older, as a proxy for adolescence and adult socioeconomic circumstances. Gender effects in socioeconomic inequalities were also examined by including gender*SEP interaction.

### Supplemental analyses

The main effects of socioeconomic position on hospitalization were additionally tested using parental income and education (not available for G1), and adult education within G2 (see Supplementary Table 3 for details).

There were too few cases of hospitalization to test inter-cohort differences in each individual condition or groups conditions. However, we tested inter-generation differences between parents (G1) and their children (G2) in age and gender adjusted models, with individual conditions as outcomes. Likewise, inter-generation differences in socioeconomic inequalities in each individual condition or groups conditions were also tested—by including generation*SEP interaction.

## Results

### Burden of hospitalization

**Fig. 2:**
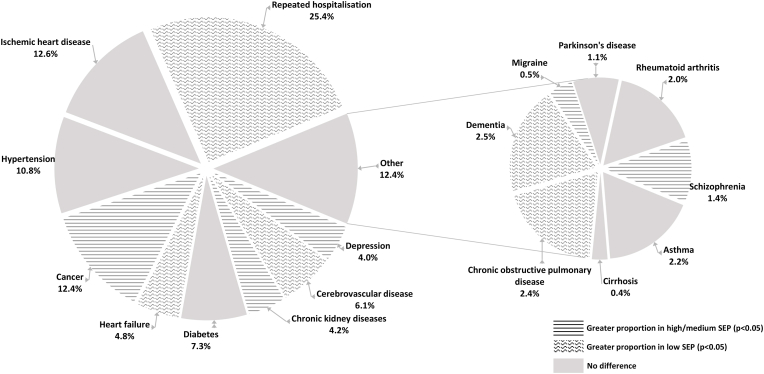
Proportion of total burden of hospitalization due to specific conditions or group of conditions. *Wavy lines indicate a greater proportion of total hospitalization due to a given conditions among those with low SEP, whereas horizontal lines show a greater proportion among those in medium/high SEP (at p < 0.05 according to chi square test).

There were 11,996 individuals who experienced at least one hospitalization during the study period—amounting to 16,073 cases of hospitalization. Fig. 2 presents the proportion of hospitalization due to each condition or group of conditions out of studied hospitalization cases. When an individual was hospitalized due to more than one condition within a five-year period—this is referred to as repeated hospitalization. The greatest proportion of cases were hospitalized due to repeated hospitalization (25.4%). Other most common reasons for hospitalization were ischemic heart disease (12.6%), cancer (12.4%), and hypertension (10.8%). Among those with low SEP—compared with high or medium—we found a greater proportion of the total burden of hospitalization being due to repeated hospitalization (absolute difference: 5.2%), dementia (1.1%), cerebrovascular disease (0.8%), heart failure (0.8%), chronic obstructive pulmonary disease (0.6%); and a lower proportion due to cancer (2.0%), depression (1.8%), chronic kidney diseases (1.4%), schizophrenia (1.5%), and migraine (0.6%).

### Cohort differences in the rate of hospitalization

**Fig. 3:**
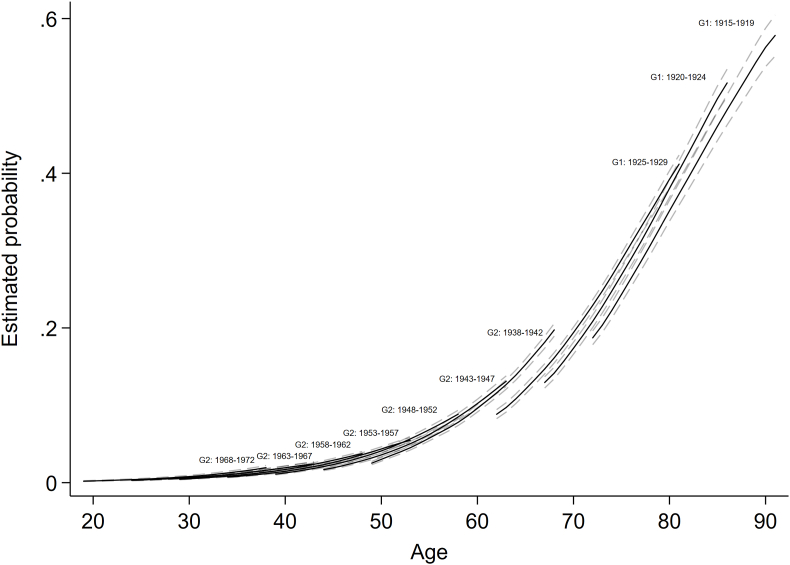
Birth-cohort-specific age trajectories of hospitalization with 95% confidence intervals represented by dashed lines.

As shown by Table 2 and Fig. 3, younger cohorts had a higher prevalence of hospitalization at overlapping ages, with inter-cohort differences emerging from early-adulthood and minimally decreasing with age in relative terms (age*YoB interaction: OR = 0.9995, 95% CI 0.9993, 0.9996), however increasing in absolute values. For instance, at age 40 predicted probability of hospitalization increased across birth-cohorts—from 1.2% (born in 1948-52) to 2.0% (born in 1963-67), whereas at age 50 it was 2.9% for those born in 1938-42 compared with 4.6% among participants born in 1953-57. At older age, the absolute cohort differences were much larger. For instance, at age 80 they increased from 33.7% to 39.0% between 1915–19 and 1925-29 birth-cohorts. Men appeared to have a higher probability of hospitalization both in relative and absolute terms, with gender differences increasing slightly across cohorts when age was held constant (gender*YoB interaction: OR = 1.01, 95% CI 1.01, 1.02, p < 0.001).

**Table 2:** Associations of age, year-of-birth with odds of hospitalization, including gender modification effects – results from the multilevel logit models.

Exposure	Cohort differences (OR 95% CI)	Cohort differences
		+ gender inequalities (OR 95% CI)
Fixed effects		
Intercept	0.04 (0.03, 0.04)	0.04 (0.04, 0.04)
Age	1.14 (1.14, 1.15)	1.14 (1.14, 1.15)
Year-of-birth (YoB)	1.04 (1.04, 1.05)	1.04 (1.03, 1.04)
Age*YoB	0.9995	0.9995
	(0.9993, 0.9997)	(0.9993, 0.9996)
Woman		0.84 (0.79, 0.90)
Woman*YoB		1.01 (1.01, 1.02)
Random effects		
Level 2: individual (intercept)	2.20 (2.03, 2.36)	2.50 (2.36, 2.64)
Level 3: family (intercept)	0.30 (0.23, 0.40)	0.02 (0.01, 0.02)
Model fit		
DIC	56988.25	56948.60
Observations	103,262	103,262

### Socioeconomic inequalities in the rate of hospitalization

**Fig. 4:**
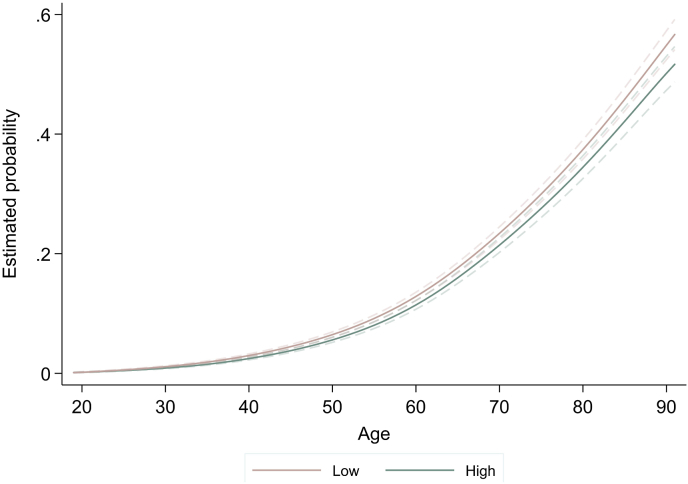
Year-of-birth adjusted age trajectories of hospitalization stratified by high vs low parental SEP.

Those born to parents with medium and low SEP had respectively 13.0% and 20.0% higher odds of experiencing hospitalization during the observation period—when age, year-of-birth and gender were accounted for. We found no evidence for differential relative socioeconomic gap in hospitalization across birth cohorts, age or gender (see Table 3)—indicating similar age slopes across these variables. However, the absolute SEP differences in hospitalization did increase across age—with rising overall prevalence (Fig. 4). For instance, at age 40 those with low SEP had 1.6% probability of hospitalization compared with 2.3% for high SEP, at age 60 this difference increased from 9.9% to 11.1% and at age 80 from 32.2% to 37.0%. Overall, consistent results were obtained when using adult education (see Supplemental Table 4 for estimates), with a somewhat stronger effect size compared with parental SEP. For instance, those with low education had 2.08 times (95% CI 1.79, 2.42) higher odds of hospitalization. There was also some evidence for an increased gap across birth cohorts between those with low compared with high education (low education*YoB interaction: OR = 1.04, 95% CI 1.02, 1.06, p < 0.001).

**Table 3:** Socioeconomic inequalities in hospitalization, including cohort, age and gender effects – results from the multilevel logit models.

Exposure	Socioeconomic inequalities OR (95% CI)	Socioeconomic inequalities	Socioeconomic inequalities	Socioeconomic inequalities
		+ cohort effects OR (95% CI)	+ age effects OR (95% CI)	+ gender effects OR (95% CI)
Fixed effects				
Intercept	0.04 (0.03, 0.04)	0.04 (0.03, 0.04)	0.04 (0.03, 0.04)	0.04 (0.03, 0.04)
Age	1.14 (1.13, 1.15)	1.14 (1.14, 1.15)	1.14 (1.14, 1.15)	1.14 (1.14, 1.15)
Year-of-birth (YoB)	1.04 (1.04, 1.05)	1.04 (1.04, 1.05)	1.04 (1.04, 1.05)	1.04 (1.04, 1.05)
Age*YoB	0.9995	0.9995	0.9995	0.9995
	(0.9994, 0.9997)	(0.9993, 0.9997)	(0.9993, 0.9997)	(0.9993, 0.9997)
Woman	0.77 (0.72, 0.82)	0.77 (0.72, 0.82)	0.77 (0.72, 0.82)	0.82 (0.73, 0.92)
Woman*YoB				
Parental SEP (high – reference)				
Medium	1.14 (1.05, 1.24)	1.12 (1.01, 1.23)	1.13 (1.01, 1.25)	1.24 (1.09, 1.43)
Low	1.21 (1.12, 1.31)	1.21 (1.10, 1.32)	1.23 (1.13, 1.35)	1.25 (1.12, 1.39)
Medium*YoB		1.00 (1.00, 1.01)		
Low*YoB		1.01 (1.00, 1.01)		
Medium*age			1.00 (0.99, 1.01)	
Low*age			1.00 (0.99, 1.00)	
Medium*woman				0.93 (0.79, 1.08)
Low*woman				0.82 (0.68, 0.99)
Random effects				
Level 2: individual (intercept)	2.47 (2.31, 2.63)	2.32 (2.10, 2.52)	2.14 (1.93, 2.34)	2.13 (1.97, 2.30)
Level 3: family (intercept)	0.0003 (0.0001, 0.0004)	0.14 (0.07, 0.26)	0.35 (0.27, 0.43)	0.35 (0.26, 0.45)
Model fit				
DIC	56508.20	56494.46	56450.29	56469.48
Observations	102,571	102,571	102,571	102,571

### Supplemental analyses

Socioeconomic inequalities in hospitalization were also found due to parental education and parental income for generation 2 (G2) (see Supplementary Table 3 for more details).

Generation 2 (G2) had higher odds of repeated hospitalization as well as hospitalization due to depression, COPD, cancer, hypertension, migraine, and rheumatoid arthritis than their parents (see Supplementary Figure 3). On the other hand, they experienced lower odds of asthma, diabetes, ischemic heart disease. No inter-generation differences were found in dementia, cerebrovascular disease, chronic kidney disease, and heart failure.

Overall, there was a trend for greater inequality in most conditions within generation 2 (G2) compared to their parents (G1). However, generation*SEP interaction did not reach significance (p < 0.05) for any of the individual conditions apart from diabetes (p = 0.03). The confidence intervals around the estimates of social inequality tended to overlap across generations as they were relatively wide (see Supplementary Figure 4). Most substantial relative inter-generation difference in SEP inequality were found for: depression (G1: OR = 0.86, 95% CI 0.56, 1.31 vs G2: OR = 1.25, 95% CI 0.98, 1.58), asthma (G1: OR = 1.16, 95% CI 0.72, 1.73 vs G2: OR = 1.41, 95% CI 0.96, 2.08), cerebrovascular disease (G1: OR = 0.98, 95% CI 0.81, 1.19 vs G2: OR = 1.39, 95% CI 1.02, 1.90), diabetes (G1: OR = 1.03, 95% CI 0.76, 1.39 vs G2: OR = 1.74, 95%CI 1.28, 2.38) (see Supplementary Figure 4).

## Discussion

### Main findings

Using the first (born 1915–1929) and second (1938–1972) generations of the Uppsala Birth Cohort Multigenerational Study, we examined cohort differences in age trajectories of hospitalization due to non-communicable conditions, and if these vary by parental socioeconomic position. In addition, we explored gender effects in cohort and socioeconomic differences in hospitalization. As hypothesized, younger cohorts had a higher prevalence of hospitalization at overlapping ages, with inter-cohort differences increasing with age. Consistent with our hypothesis, those born to parents with medium and low socioeconomic position had higher odds of experiencing hospitalization during the observation period, with the socioeconomic gradient remaining stable across cohorts. We found no evidence for varying socioeconomic differences in hospitalization across age or gender in relative terms. However, due to the overall increase in the probability of hospitalization with age,the absolute difference between low and high SEP of parents increased as participants got older.

### Comparison with other evidence

Our findings are consistent with a study conducted in the Netherlands, investigating trends in primary care visits due to major non-communicable conditions, which showed an increase in visits by nearly 6% between 2004 and 2011 (van Oostrom et al., 2016). However, this study explored age-adjusted period trends, as opposed to differences across birth cohorts. The evidence from this study is in line with previous findings from Sweden as well as other high-income countries, which found increasing burden of non-communicable morbidity between 1989 and 2008, when ageing was accounted for (Andersson et al., 2015; Fors et al., 2008; Gondek et al., 1946; Meinow et al., 2015; Paren et al., 2014; Parker & Thorslund, 2007). However, contrary evidence also exists indicating an increase in healthy life expectancy (Salomon et al., 2012) and years free from disability and mobility problems from early 1990s until early 2010s in Sweden (Sundberg et al., 2016).

As in our study, the evidence consistently shows that those in lower socioeconomic position—defined according to social class, education or income—had worse health (Fors et al., 2008; Fritzell et al., 2007; Kunst et al., 2005; Lahelma et al., 2002; Meinow et al., 2015) and were more likely to be admitted to a hospita across most high-income countries (Barnett & Lauer, 2003; Brameld & Holman, 2005; Carlsen et al., 2007; Lofqvist et al., 2014). As found by previous studies as well as our research, the social gradient has remained stable, in relative terms, from 1990s to 2000s in Sweden (Fritzell et al., 2007; Hu et al., 2016; Kunst et al., 2005; Lahelma et al., 2002). Our study is the first one, however, which compared trajectories in hospitalization due to major non-communicable conditions across such a long range of birth-cohorts (1915–1929; 1938–1972) and age (19–91), which helps to disentangle age and cohort effects in this health outcome. This contributes to the literature by showing that inter-cohort differences as well as the socioeconomic gradient in health can be already observed in early adulthood and started to emerge among cohorts born as early as the beginning of the 20th century. Previous longitudinal evidence in Sweden was limited to older population (Fors et al., 2008; Meinow et al., 2015; Parker & Thorslund, 2007), whereas most life course studies come from the UK—showing higher rates of frailty and risk factors for morbidity, such as BMI and blood pressure, in younger cohorts (Bann et al., 2017; Marshall et al., 2015; Wills et al., 2011).

### Interpretation of the findings

An increase in hospitalization across younger birth cohorts may be due to rising burden of non-communicable morbidity. If the burden of non-communicable morbidity among people of the same age but born at a later time, is increasing—this leads to extending their lifespan with morbidity. This provides support for the scenario known as expansion of morbidity (Gruenberg, 1977). However, the relationship between morbidity and hospitalization is complex and potential reasons for an increase in hospitalization may be related to both changes over time in the prevalence of morbidity and healthcare utilization.

If increasing hospitalization is driven by rising morbidity, there are several complementary explanations for worsening health across birth-cohorts. The trends in non-communicable conditions could be explained by changes over time in risk factors. However, these trends are inconsistent, with certain risk factors decreasing since 1990s—e.g. hypertension (Ng et al., 2012), smoking (Patja et al., 2009), or alcohol consumption (Heckley et al., 2017) and others increasing—e.g. fat intake (Johansson et al., 2012), cholesterol (Johansson et al., 2012), and obesity (Johansson et al., 2012). Another hypothesis is that more effective disease management appears to have led to improved survival with previously fatal diseases (Davies et al., 2011; Simpson et al., 2005; Simpson & Sheikh, 2010). According to this scenario, known as expansion of morbidity, we observe a greater number of people and extended lifespan with major non-communicable conditions associated with ageing, such as cancer or coronary heart disease—resulting from reduced mortality due to those disorders. (Barasa et al., 2014; Moller et al., 2003). Improved management of non-communicable conditions may have also led to the conditions being less disabling over time. For instance, one study found an increase in years free from disability and mobility problems from early 1990s until early 2010s in both elderly men and women in Sweden (Sundberg et al., 2016).

It is also likely that increases in diagnosed and treated conditions are due to more accessible healthcare services, improved quality of healthcare and more common screening (e.g. of cancer, diabetes or asthma) (McKee et al., 2020). However, since the early 1990s the effort has been made to care for elderly individuals as far as possible in their own homes or within primary healthcare, which aimed to reduce the hospital visits (Marczewska, 2011).

Other potential reasons for rising hospitalization may be a greater health awareness and propensity to seek help, which are associated with rising education over time (Johansson et al., 2012). However, a greater inclination to seek help or report health problems still indicate a growing demand on health services and should be considered as a public health problem as opposed to an artefact in the data on secular trends.

The rising trend in hospitalization may not be universal across all major non-communicable conditions. Age-cohort investigation of trends in each health condition was not feasible in this study due to hospitalization being a relatively rare outcome. However, we compared the prevalence of hospitalization between parents (born in 1915–1929) and their children (1938–1972) accounting for age differences. We found that the younger generation had a greater prevalence of hospitalization due to such conditions as depression, COPD, hypertension and migraine. These conditions are typically managed in ambulatory care and hospitalization is considered to be potentially avoidable (Bardsley et al., 2012). On the contrary, hospitalization due to asthma and diabetes did decrease over time indicating an improvement in patient management and access to primary care, despite some evidence for their rising prevalence (Backman et al., 2017; Jansson et al., 2015).

We found a greater prevalence of hospitalization due to non-communicable conditions among those born to parents of a disadvantaged social class. This may be due to overall worse health among those in disadvantaged social circumstances (Fors et al., 2008; Fritzell et al., 2007; Kunst et al., 2005; Lahelma et al., 2002; Meinow et al., 2015). It is also likely that individuals with a lower social class are at a higher risk of clustering and accumulating risk factors for morbidity—such as poor diet, smoking, physical inactivity or engaging in risky behaviors (Dannefer, 2003). However, a higher prevalence of morbidity does not need to directly translate into higher demands for healthcare services (Burstrom, 2009). Factors such as healthcare-seeking, communication skills, health literacy and practices may all play an important role in inequalities in hospitalization. It would be beneficial to further understand to what extent these contextual factors and health needs explain higher rates of hospitalization in more disadvantaged groups.

We found that no progress was made in reducing the socioeconomic inequalities in hospitalization due to non-communicable conditions from 1989 to 2008. This is despite Sweden having been particularly determined to reduce the health gap between rich and poor, making it a central objective of public health and social policy agendas since 1980s (Lundberg, 2018). Furthermore, there was an indication of greater inequality among the younger generation compared to their parents in number of conditions, due to which hospitalization could be avoided. This includes depression, asthma, diabetes or heart failure. It is important to understand the reasons for this increase in inequity in healthcare utilization to facilitate devising more effective policies and interventions.

### Strengths and limitations

The main strength of the current study is the use of register databases, which enable analysis with virtually no missing data for other reasons than emigration or death. In addition, the data are free from response biases, such as recall or social desirability, which are present in self-reported outcomes. Moreover, the registries allow for setting up the data as an accelerated longitudinal study—which is a gold standard for studying age and cohort effects. Nonetheless, there is no guarantee that cohort effects are definitely not attributable to period effects. Period effects could be manifested by an overall trend in the population to have a greater propensity to report health problems, for instance, due to rising health awareness and expectations. This, however, would still translate into higher rates of hospitalization in younger cohorts at the same age—captured by age-cohort analysis. In order to have a ubiquitous influence on the entire population, periodical factors would need to be extreme in nature—for instance resulting from famine or war. However, these events are well-documented and are likely to result in short-term fluctuations in morbidity trends (e.g. 2nd World War, Spanish flu, The Dutch famine of 1944–45). Another limitation of the study is that the data were available only until 2008, hence studying the trends during the last decade was not possible.

## Conclusion

Younger cohorts had a higher prevalence of hospitalization at overlapping ages, with inter-cohort differences increasing with age in absolute terms due to the overall rising probability of hospitalization. Those born to parents with medium and low socioeconomic position had higher odds of experiencing hospitalization during the observation period—with no evidence of reductions in the socioeconomic gradient across cohorts. Our findings emphasize a need for policies and interventions reducing the burden of non-communicable morbidity, particularly among those of low socioeconomic position.

## Funding

Dawid Gondek is funded by the 10.13039/501100000269Economic and Social Research Council (10.13039/501100000269ESRC +3 PhD Studentship). This work was supported by the 10.13039/501100004359Swedish Research Council (project 2013–05104), the 10.13039/501100006636Swedish Research Council for Health, Working Life and Welfare (project 2018–00211) and the Ageing Trajectories of Health: Longitudinal Opportunities and Synergies (ATHLOS) project. The ATHLOS project received funding from the European Union's 10.13039/501100007601Horizon 2020 research and innovation program under grant agreement No 635316. The funding bodies had no role in the design of the study, analysis and interpretation of data, and in writing the manuscript.

## Ethical approval

The study was approved by the Regional Ethics board in Stockholm.

## Informed consent

Informed consent was obtained from all individual participants included in the study.

## Data availability statement

The data are available at the Centre for Health Equity Studies (CHESS).

## CRediT authorship contribution statement

**Dawid Gondek:** Conceptualization, Methodology, Software, Formal analysis, Data curation, Writing - original draft, Visualization. **George B. Ploubidis:** Conceptualization, Methodology, Writing - review & editing, Supervision. **Muhammad Zakir Hossin:** Validation, Writing - review & editing, Conceptualization. **Menghan Gao:** Writing - review & editing, Conceptualization. **David Bann:** Writing - review & editing. **Ilona Koupil:** Conceptualization, Methodology, Software, Resources, Data curation, Supervision, Project administration, Funding acquisition.

## Declaration of competing interest

The authors declare that they have no conflict of interest.
