# Supplementary material for: Inequality in hospitalization due to non-communicable diseases in Sweden: Age-cohort analysis of the Uppsala Birth Cohort Multigenerational Study
Source: SSM Popul Health. 2021 Jan 21;13:100741. doi: 10.1016/j.ssmph.2021.100741 (PMC7841359; doi:10.1016/j.ssmph.2021.100741)
Supplement: Multimedia component 1 [file mmc1.docx]

**Supplementary Table 1.** Predictors of being excluded due to death or emigration.

**Supplementary Table 2.** Conditions comprising the outcome variable and corresponding ICD-9/10 codes.

**Supplementary Figure 1.** Visualization of the data structure.

**Supplementary Figure 2.** Visualization of hierarchical data structure.

**Supplementary Table 3.** Odds ratio with confidence intervals for the association between the exposures and hospitalization. The most parsimonious models presented – with significant covariates only (at p<0.05) and best goodness-of-fit.

**Supplementary Figure 3.** Relative difference between children and parents (a reference group) in odds of hospitalization due to individual conditions/group of conditions.

**Supplementary Figure 4.** Relative difference between low and medium/high SES (a reference group) in odds of hospitalization due to individual conditions/group of conditions across both generations.

| Supplementary Table 1. Predictors of being excluded due to death or emigration. | | | | |
| --- | --- | --- | --- | --- |
|  |  | Died or emigrated before 1994 |  | Died or emigrated in 1994-2008 |
|  |  | Odds ratio (95% CI)* |  | Odds ratio (95% CI)** |
| Female |  | 0.62 (0.58, 0.66) |  | 0.66 (0.60, 0.73) |
| Parental SEP (high – reference) |  |  |  |  |
| Medium |  | 1.64 (1.48, 1.81) |  | 4.04 (3.49, 4.69) |
| Low |  | 2.61 (2.08, 2.46) |  | 6.67 (5.91, 7.68) |
| Hospitalized in 1989-1993 |  |  |  | 3.79 (3.55, 4.06) |
| *Estimates from logit model: n=32,448-32,767. This sample includes individuals who did not contribute any data to the analyses.  **Estimates from multilevel logit model: n=28,238-28,448 (observations: n=112,952-113,792). This sample includes only individuals who contributed to at least one observation period. | | | | |

| Supplementary Table 2. Conditions comprising the outcome variable and corresponding ICD10/9 codes. ‘X’ corresponds to any number. | | |
| --- | --- | --- |
| **Condition** | **ICD-10** | **ICD-9** |
| Depression^(1)^ | F20.4, F31.3, F31.4, F31.5, F32, F33, F34.1, F41.2, F43.2 | 296.2, 296.3, 296.5, 300.4, 309, 311 |
| Asthma^(2)^ | J45 | 493 |
| Stroke or transient ischemic attack^(3)^ | G45.0, G45.1, G45.2, G45.3, G45.8, G45.9, H34.1, I60, I61, I63, I64 | 362.3, 430, 431, 433.x1, 434.x1, 435, 436 |
| Chronic kidney diseases^(4)^ | N01, N02, N03, N04, N05, N06, N07, N08, N10, N11, N12, N13, N14, N15, N16, N17, N18, N19, N20, N21, N22, N23 | 583, 584, 585, 586, 592, 593.9 |
| **Chronic liver disease^(5)^** | K70.0, K70.2, K73.X, K754, K758, K75.9, K76.0, B18.0, B18.1, B18.2, B18.8, B18.9 | 070.2X, 070.3X, 070.4X, 070.5X, 070.6, 070.9, 571.0, 571.3, 571.4X, 571.8, 573.1, 573.3 |
| Chronic obstructive pulmonary disease^(6)^ | J40, J43.0, J43.1, J43.2, J43.8, J43.9, J44, J44.0, J44.1, J44.8, J44.9 | 492, 492.0, 492.8, 496.x |
| Dementia^(1)^ | F00, F01, F02, F03, F051, G30, G31 | 290, 294.1, 331.2 |
| Diabetes^(7)^ | E10-E14 | 250 |
| Heart failure (congestive heart failure; left ventricular failure; unspecified heart failure)^(8)^ | I50 | 428 |
| Cancer^(1, 9)^ | C18, C19, C20, C21, C33, C34, C38.4, C45.0, C46.71, C50, C53, C61, C77, C78, C79, C80, C81, C82, C83, C84, C85, C88, C90.0, C90.2, C96, D05, D06, D01.0, D01.3, D02.2, D07.5 | 153, 154, 162, 163, 174, 180, 185, 196, 197, 198, 199, 200, 201, 202, 203.0, 230.3, 230.4, 230.5, 230.6, 231.2, 233.0, 233.1, 233.4, 238.6 |
| Hypertension^(10)^ | I10-I15 | 401-405 |
| Coronary heart disease^(8)^ | I20–I25 | 410-414 |
| Migraine^(11)^ | G43 | 346 |
| Parkinson’s disease^(12)^ | G20-G22 | 332 |
| Rheumatoid arthritis^(1)^ | M05, M06, M31.5, M32, M33, M34, M35.1, M35.3, M36.0 | 446.5, 710.0, 710.1, 710.2, 710.3, 710.4, 714.0, 714.1, 714.2, 714.8, 725 |
| Schizophrenia or schizoaffective disorder^(13)^ | F20, F21, F25 | 295 |

Supplementary Figure 1. Visualization of the data structure.


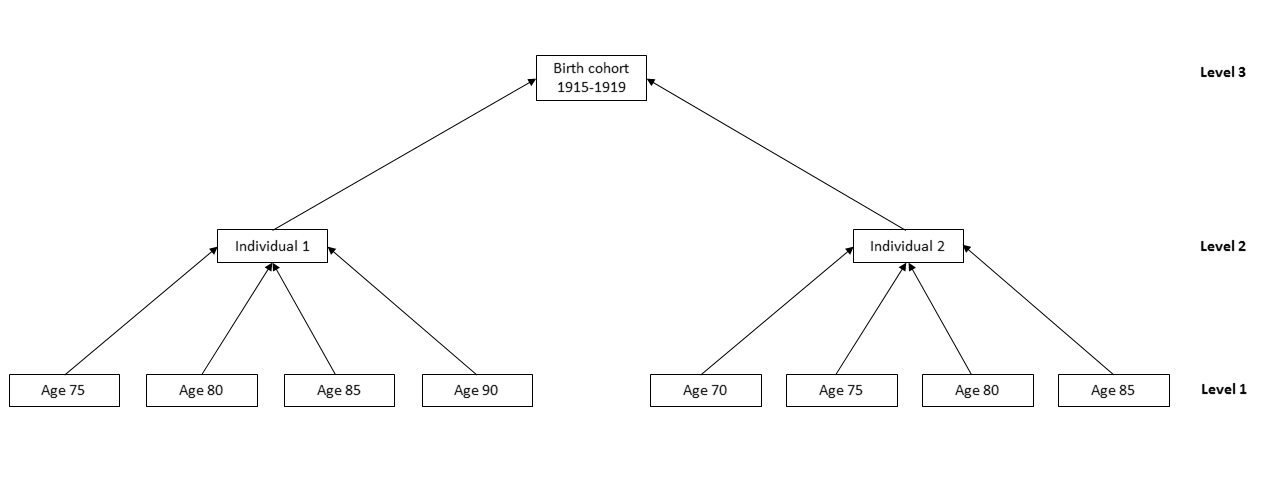


Supplementary Figure 2. Visualization of hierarchical data structure.

| Supplementary Table 3. Odds ratio with confidence intervals for the association between the exposures and hospitalization in the second generation only. The most parsimonious models presented – with significant covariates only (at p<0.05). | | | | |
| --- | --- | --- | --- | --- |
|  | Parental SEP^a^ | Parental education^b^ | Parental income^c^ | Adult education^d^ |
| **Fixed effects** |  |  |  |  |
| Intercept | 0.01 (0.01, 0.02) | 0.01 (0.01, 0.01) | 0.02 (0.01, 0.02) | 0.01 (0.01, 0.01) |
| Age | 1.13 (1.13, 1.14) | 1.13 (1.12, 1.14) | 1.13 (1.12, 1.14) | 1.13 (1.13, 1.14) |
| Year-of-birth (YoB) | 1.02 (1.01, 1.03) | 1.02 (1.01, 1.04) | 1.02 (1.01, 1.03) | 1.03 (1.02, 1.04) |
| Female | 0.89 (0.81, 0.98) | 0.89 (0.80, 0.99) | 0.88 (0.80, 0.96) | 0.92 (0.83, 1.01) |
| Parental SEP (high – reference) |  |  |  |  |
| Medium | 1.10 (0.96, 1.25) |  |  |  |
| Low | 1.24 (1.11, 1.36) |  |  |  |
| Parental education (high – reference) |  |  |  |  |
| Medium |  | 1.24 (1.07, 1.47) |  |  |
| Low |  | 1.43 (1.24, 1.68) |  |  |
| Parental income |  |  | 0.90 (0.85, 0.95) |  |
| Adult education (high – reference) |  |  |  |  |
| Medium |  |  |  | 1.42 (1.23, 1.63) |
| Low |  |  |  | 2.02 (1.72, 2.31) |
| **Random effects** |  |  |  |  |
| Level 2: individual (intercept) | 3.20 (2.79, 3.51) | 3.35 (3.06, 3.69) | 3.35 (3.10, 3.61) | 3.28 (2.98, 3.57) |
| Level 3: family (intercept) | 0.15 (0.07, 0.36) | 0.02 (0.01, 0.04) | 0.005 (0.002, 0.0012) | 0.0003 (0.002, 0.0003) |
| **Model fit** |  |  |  |  |
| DIC | 27531.84 | 27526.32 | 27465.85 | 27486.95 |
| **Observations** | 75,342 | 75,342 | 75,090 | 75,092 |
| ^a^Socioeconomic position (SEP) was defined in the same way as in the main manuscript (see Table 1).  ^b^A categorical variable (0 = High, 1 = Medium, 2 = Low), with categories grouped as: low (compulsory schooling), intermediate (upper-secondary schooling) and high (any postsecondary education). Parental education was measured as the highest lifetime education of either parent from Census and Education register in 1960-2008.  ^c^A continuous individual disposable income standardized by age and gender in each calendar year (obtained from LOUISE and LISA registers 1990-2008, and Census 1970-1990), and then averaged across all available calendar years, and both parents, when the parents were aged 25-65, and was obtained from Censuses 1970 and 1990 and from the LISA 1990–2008.  ^d^ Adult education was measured at age 19 or older and it was classified as high (post-secondary ≥3 years; post-graduate), medium (upper secondary; post-secondary <3 years) and low (<upper secondary). | | | | |

| Supplementary Table 4. Socioeconomic inequalities (according to education in adulthood^a^) in hospitalization, including cohort, age and gender effects – results from the multilevel logit models. | | | | |
| --- | --- | --- | --- | --- |
| Exposure | Socioeconomic inequalities | Socioeconomic inequalities  + cohort effects | Socioeconomic inequalities  + age effects | Socioeconomic inequalities  + gender effects |
| **Fixed effects** |  |  |  |  |
| Intercept | 0.02 (0.01, 0.02) | 0.02 (0.02, 0.02) | 0.02 (0.01, 0.02) | 0.02 (0.01, 0.02) |
| Age | 1.10 (1.09, 1.11) | 1.10 (1.09, 1.11) | 1.10 (1.09, 1.13) | 1.10 (1.09, 1.11) |
| Year-of-birth (YoB) | 1.10 (1.09, 1.11) | 1.09 (1.06, 1.10) | 1.10 (1.09, 1.11) | 1.10 (1.09, 1.11) |
| Age*YoB | 1.01 (1.01, 1.01) | 1.01 (1.01, 1.01) | 1.01 (1.01, 1.01) | 1.01 (1.01, 1.01) |
| Woman | 0.91 (0.83, 1.00) | 0.92 (0.83, 1.00) | 0.91 (0.83, 1.00) | 0.92 (0.73, 1.13) |
| Woman*YoB |  |  |  |  |
| Education^a^ (high – reference) |  |  |  |  |
| Medium | 1.47 (1.29, 1.69) | 1.39 (1.14, 1.66) | 1.44 (1.24, 1.70) | 1.47 (1.19, 1.73) |
| Low | 2.08 (1.79, 2.42) | 1.74 (1.42, 2.08) | 2.01 (1.70, 2.38) | 2.18 (1.76, 2.61) |
| Medium*YoB |  | 1.01 (0.99, 1.03) |  |  |
| Low*YoB |  | 1.04 (1.02, 1.06) |  |  |
| Medium*age |  |  | 1.00 (0.98, 1.01) |  |
| Low*age |  |  | 0.98 (0.97, 1.00) |  |
| Medium*woman |  |  |  | 0.92 (0.68, 1.20) |
| Low*woman |  |  |  | 1.02 (0.78, 1.36) |
| **Random effects** |  |  |  |  |
| Level 2: individual (intercept) | 3.16 (2.85, 3.48) | 3.35 (3.06, 3.65) | 3.29 (3.02, 3.56) | 3.29 (3.02, 3.56) |
| Level 3: family (intercept) | 0.15 (0.05, 0.28) | 0.02 (0.01, 0.03) | 0.0002 (0.0002, 0.0003) | 0.0002 (0.0002, 0.0003) |
| **Model fit** |  |  |  |  |
| DIC | 27998.07 | 27951.60 | 28005.36 | 28008.22 |
| **Observations** | 83364 | 83364 | 83364 | 83364 |
| ^a^Education was measured at age 19 or older and it was classified as high (post-secondary ≥3 years; post-graduate), medium (upper secondary; post-secondary <3 years) and low (<upper secondary). | | | | |


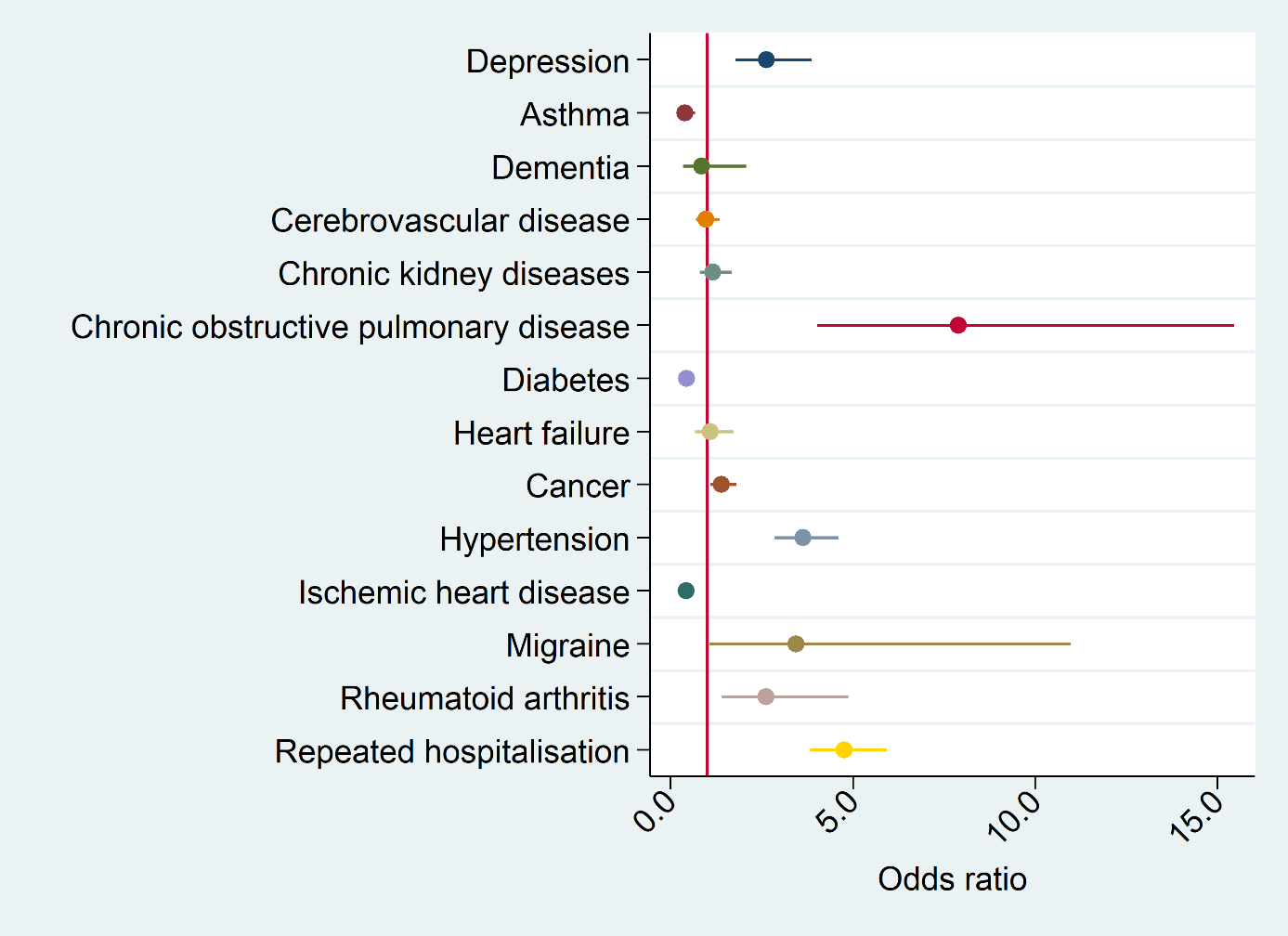


Supplementary Figure 3. Relative difference between children and parents (a reference group) in odds of hospitalization due to individual conditions/group of conditions.


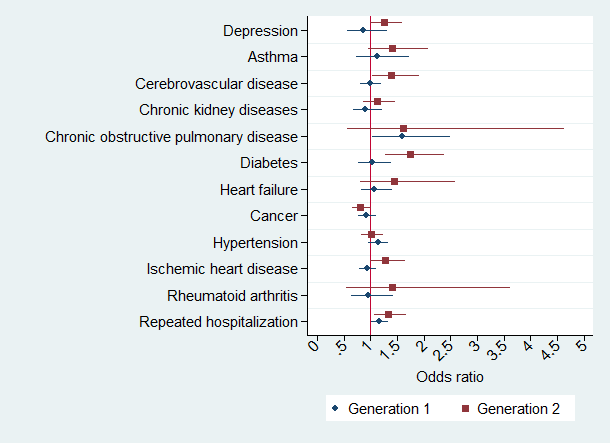


Supplementary Figure 4. Relative difference between low and medium/high SES (a reference group) in odds of hospitalization due to individual conditions/group of conditions across both generations.

**References**

1. Quan H, Li B, Saunders LD, Parsons GA, Nilsson CI, Alibhai A, et al. Assessing validity of ICD-9-CM and ICD-10 administrative data in recording clinical conditions in a unique dually coded database. Health Serv Res. 2008;43(4):1424-41.

2. Gershon AS, Wang C, Guan J, Vasilevska-Ristovska J, Cicutto L, To T. Identifying patients with physician-diagnosed asthma in health administrative databases. Can Respir J. 2009;16(6):183-8.

3. Kokotailo RA, Hill MD. Coding of stroke and stroke risk factors using international classification of diseases, revisions 9 and 10 Stroke. 2005;36(8):1776–81.

4. Ronksley PE, Tonelli M, Quan H, Manns BJ, , , James MT, Clement FM, et al. Validating a case definition for chronic kidney disease using administrative data. Nephrol Dial Transplant. 2012;27(5):1826–31.

5. Lapointe-Shaw L, Georgie F, Carlone D, Cerocchi O, Chung H, Dewit Y, et al. Identifying cirrhosis, decompensated cirrhosis and hepatocellular carcinoma in health administrative data: A validation study. PLoS One. 2018;13(8):e0201120.

6. Zhang Q, Bush K, Nolan J, Schnier C, Sudlow C. Definitions of Chronic Obstructive Pulmonary Disease for UK Biobank Phase 1 Outcomes Adjudication. UK Biobank; 2008.

7. Hux JE, Ivis F, Flintoft V, Bica A. Diabetes in Ontario: determination of prevalence and incidence using a validated administrative data algorithm. Diabetes Care. 2002;25(3):512–6.

8. Murray CJ, Kulkarni SC, Ezzati M. Understanding the coronary heart disease versus total cardiovascular mortality paradox: a method to enhance the comparability of cardiovascular death statistics in the United States. Circulation. 2006;113(17):2071-81.

9. Penberthy L, McClish D, Pugh A, Smith W, Manning C, Retchin S. Using hospital discharge files to enhance cancer surveillance. Am J Epidemiol. 2003;158(1):27-34.

10. Quan H, Khan N, Hemmelgarn BR, Tu K, Chen G, Campbell N, et al. Validation of a case definition to define hypertension using administrative data. Hypertension. 2009;54(6):1423–8.

11. Vosoughi K, Stovner LJ, Steiner TJ, Moradi-Lakeh M, Fereshtehnejad SM, Farzadfar F, et al. The burden of headache disorders in the Eastern Mediterranean Region, 1990-2016: findings from the Global Burden of Disease study 2016. J Headache Pain. 2019;20(1):40.

12. Noyes K, Liu H, Holloway R, Dick AW. Accuracy of Medicare claims data in identifying Parkinsonism cases: comparison with the Medicare current beneficiary survey. Mov Disord. 2007;22(4):509-14.

13. Stewart CC, Lu CY, Yoon TK, Coleman KJ, Crawford PM, Lakoma MD, et al. Impact of ICD-10-CM Transition on Mental Health Diagnoses Recording. EGEMS (Wash DC). 2019;7(1):14.
